# Tuberculous Enteritis: A Rare Complication of Miliary Tuberculosis

**DOI:** 10.1155/2016/6949834

**Published:** 2016-02-28

**Authors:** Danisha Figueroa, Nilmarie Guzman, Carmen Isache

**Affiliations:** Department of Internal Medicine, Division of Infectious Disease, University of Florida College of Medicine-Jacksonville, Jacksonville, Fl 32209, USA

## Abstract

Tuberculous enteritis is a clinical rarity even in immunocompromised patients. We present a case of miliary tuberculosis with gastrointestinal involvement. A 47-year-old homosexual male from Philippines with no significant medical history presented with productive cough, night sweats, subjective fevers, shortness of breath, watery diarrhea, and 25-pound weight loss in past one year. On physical exam he was afebrile, mildly hypotensive, tachycardic, and tachypneic, but saturating well on room air. He was cachectic with oral thrush and bilateral fine rales. Chest X-ray revealed a miliary pattern. His sputum AFB smear was strongly positive. PCR and sputum culture were positive for* Mycobacterium tuberculosis*. He was started on Rifampin, Isoniazid, Ethambutol, and Pyrazinamide. He was found to be HIV positive with an absolute CD4 count of 4 cells/*μ*L. Due to persistent diarrhea, stool was sent for AFB culture and grew* M. tuberculosis*. He responded well to treatment with resolution of symptoms. Tuberculous enteritis occurs in about 2% of the patients with pulmonary tuberculosis. Although it is uncommon, it should be considered in patients with active pulmonary tuberculosis and abdominal complaints. A presumptive diagnosis of tuberculous enteritis can be made in the setting of active pulmonary tuberculosis with suggestive clinical, endoscopic, and/or radiographic findings.

## 1. Background

Intestinal tuberculosis is difficult to discriminate from other intestinal diseases due to its nonspecific symptoms but must be kept in the differential in patients diagnosed with pulmonary tuberculosis and gastrointestinal symptoms.

A presumptive diagnosis of tuberculous enteritis can be made in the setting of known active pulmonary tuberculosis, with clinical, endoscopic, and/or radiographic findings suggestive of intestinal tuberculosis.* M. tuberculosis* usually invades the ileocecal region due to its relative stasis and abundant lymphoid tissue.

We report a case of miliary tuberculosis complicated with tuberculous enteritis in a newly diagnosed HIV/AIDS patient.

## 2. Case Report

A 47-year-old homosexual Filipino male, with no significant past medical history, presented to the hospital complaining of cough productive with white sputum for the past year. Associated symptoms included night sweats, intermittent subjective fevers, general malaise, and shortness of breath. His symptoms had been worsening for past few days. Patient was also complaining of intermittent watery diarrhea mainly with meals, abdominal pain, and 25-pound weight loss in the past year. He had never been homeless. He lived in a trailer with his mother and nephew. He reported history of incarceration 24 years before, while he was still living in the Philippines. He immigrated to United States in 1995. He denied any recent sick contacts.

On admission he was cachectic, with evidence of temporal wasting. He was in mild respiratory distress. He was afebrile with a heart rate of 93 beats per minute and tachypnea of 23 breaths per minute. He had no palpable lymphadenopathy. Severe oral thrush was present. On chest auscultation, he had bilateral fine rales. The rest of the examination was unremarkable.

Laboratory workup showed a normal white cell count, normocytic anemia, hyponatremia of 131 mmol/L, hypoalbuminemia of 2.1 grams/dL, mild elevation of aspartate aminotransferase to 75 U/L, and an elevated lactate dehydrogenase of 654. Lactic acid was normal. Bacterial and AFB blood cultures were negative. HIV test was positive. The patient was not aware of HIV status prior to this admission. His absolute CD4 count was 4 cells/*μ*L. Sputum AFB smear was strongly positive. Sputum PCR for* Mycobacterium tuberculosis* was also positive. Chest X-ray showed bilateral diffuse interstitial and airspace opacities. Computer tomography of chest with intravenous contrast showed innumerable random nodules throughout the lung parenchyma bilaterally. Some of the nodules within the upper lobes had radiologic evidence of central cavitation. Right hilar and mediastinal bulky adenopathy was seen, as well as hypodense tiny cystic lesions within the liver and spleen. Computer tomography of abdomen with intravenous contrast showed retroperitoneal, pelvic, and inguinal enlarged necrotic lymph nodes with small and large bowel unremarkable. Since patient continued to have diarrhea and abdominal pain, stool sample for AFB smear and culture was obtained. The stool sample was PCR positive for* M. tuberculosis*.

Patient was initiated on Rifampin, Isoniazid, Ethambutol, and Pyrazinamide. He responded well to therapy. His cough and diarrhea progressively improved. After 8 weeks of antituberculous therapy he was also started on antiretroviral therapy with Atripla. His sputum AFB culture was negative after two months of therapy. He was followed up in Infectious Disease Clinic as outpatient. At the end of 6 months of antituberculous therapy, he had gained 20 pounds and his cough and diarrhea had completely resolved.

## 3. Discussion

Tuberculosis (TB) is second to HIV/AIDS as the greatest killer worldwide due to a single infectious agent. According the World Health Organization, in 2013 over 9 million people were diagnosed with tuberculosis and 1.5 million died from the disease. The largest number of new tuberculosis cases occurs in the South-East Asia and Western Pacific Regions, accounting for 56% of total new cases globally [1].

Tuberculosis is a social disease with medical aspects described as barometer of social welfare. The social factors are poverty, illiteracy, ignorance, overcrowding, population explosion, undernutrition, and lack of awareness about illness [2].

Along with the increase incidence of pulmonary tuberculosis in parallel with the increase in population in various regions of the world, in recent years, the incidence of abdominal tuberculosis has also increased [3]. Abdominal tuberculosis represents the sixth most frequent form of extrapulmonary tuberculosis after lymphatic, genitourinary, bone and joint, and meningeal tuberculosis [4].

Gastrointestinal tuberculosis remains a common problem in impoverished areas of the world but is relatively infrequent in the United States [3].

Intestinal tuberculosis frequently occurs in adults with a male to female ratio of 1 : 2 [5]. It has a nonspecific presentation including chronic abdominal pain, fatigue, anorexia, diarrhea, and weight loss that can range from weeks to months. Approximately 25–50% of patients may present with a palpable right lower quadrant mass. Bowel obstruction is the most common complication. The pathogenesis is attributed to swallowing of infected sputum, hematogenous spread from active pulmonary or miliary tuberculosis, ingestion of contaminated milk or food, or contiguous spread from adjacent organs [5, 6].

Diagnosis is made in the setting of known active pulmonary tuberculosis, with clinical, endoscopic, and/or radiographic findings suggestive of intestinal tuberculosis. CT findings consist of concentric mural thickening of the ileocecal region, with or without proximal intestinal dilatation and hypodense lymph nodes with peripheral enhancement in the mesentery and retroperitoneum. Endoscopically, TB enteritis can present in three different forms: ulcerative, hypertrophic, or ulcerohypertrophic. The ulcerative type commonly affecting the ileum and jejunum is characterized by single or multiple transverse ulcers forming strictures during healing process and may perforate, bleed, or form fistulas. The hypertrophic and ulcerohypertrophic types commonly affect the ileocecum and cause obstruction or present as a mass [4]. Polymerase Chain Reaction (PCR) can be used to identify* M. tuberculosis* in blood, urine, stool, or tissue. The diagnostic rate of intestinal tuberculosis increases if an active tuberculous lesion is observed in other regions when the diagnosis is suspected and identification of a caseating granuloma is made on pathologic exam or through the detection of additional* Mycobacterium tuberculosis* cultures [5].

The most effective treatment of uncomplicated tuberculous enteritis is antituberculous therapy for total of 6 to 9 months. For the first two months, quadruple therapy is recommended. First-line drugs include Isoniazid, Rifampin, Ethambutol, and Pyrazinamide. For the subsequent four to seven months, if no resistance is identified, then dual therapy with Isoniazid and Rifampin can be used [6, 7]. The prognosis of abdominal tuberculosis is usually guarded, with an overall mortality between 19% and 38% [7].

Our patient presented with weight loss, shortness of breath, and productive cough for one year associated with significant risk factors for tuberculosis including being an immigrant from a TB endemic region, HIV infection, and history of imprisonment. A diagnosis of pulmonary tuberculosis was made based on clinical manifestations and positive sputum AFB smear and culture. In light of patient's persistent diarrhea with negative stool workup, a diagnosis of TB enteritis was suspected. Stool sample had strongly positive AFB smear and positive PCR for* M. tuberculosis*. Patient responded well to antituberculous therapy with complete resolution of his diarrhea, respiratory symptoms, and weight gain. He is currently responding well to antiretroviral therapy.

This case demonstrates that tuberculous enteritis is a significant cause of diarrhea in patients with active pulmonary tuberculosis and must be kept in the differential diagnosis when all other stool cultures are negative mainly in immunocompromised patients like with HIV with poor immunovirological control. Patients usually respond well to therapy if good compliance is achieved.
